# Epidemiology of Imported Leishmaniasis in Italy: Implications for a European Endemic Country

**DOI:** 10.1371/journal.pone.0129418

**Published:** 2015-06-26

**Authors:** Trentina Di Muccio, Aldo Scalone, Antonella Bruno, Massimo Marangi, Romualdo Grande, Orlando Armignacco, Luigi Gradoni, Marina Gramiccia

**Affiliations:** 1 Unit of Vector-borne Diseases & International Health, MIPI Department, Istituto Superiore di Sanità, Rome, Italy; 2 Laboratory of Parasitology, Unit of Microbiology and Virology, IRCCS Policlinico San Matteo, Pavia, Italy; 3 Unit of Infectious Diseases, Department of Medical Sciences, Sant’Andrea Hospital, Rome, Italy; 4 Unit of Clinical Microbiology Virology and Bioemergencies Diagnosis, Luigi Sacco University Hospital, Milan, Italy; 5 Unit of Infectious Disease, Belcolle Hospital, Viterbo, Italy; Pasteur Institute of Islamic Republic of Iran

## Abstract

In the past decade, the number of imported leishmaniasis cases has increased in countries of Western Europe. The trend is associated with increasing travels, ecotourism activity, military operations and immigration. While in endemic countries leishmaniasis is usually well diagnosed, accurate patient history and parasite identification are necessary to distinguish between autochthonous and imported cases. This is particularly important, as new *Leishmania* species/genotypes may be introduced and transmitted by local phlebotomine vectors without appropriate surveillance, with unpredictable consequences. We report on the surveillance of imported leishmaniasis performed by the *Leishmania *Identification Reference Centre of Rome from 1986 through 2012, involving health care centres from 16/20 Italian regions. Suspected imported cases were analyzed and conclusions were based on clinical, epidemiological and diagnostic findings. Over the years, different parasite identification methods were employed, including MultiLocus Enzyme Electrophoresis and molecular techniques combining disease diagnosis (SSU rDNA nested-PCR) and *Leishmania* typing (nuclear repetitive sequence and ITS-1 PCR-RFLPs). A total of 105 imported cases were recorded (annual range: 0-20) of which 36 were visceral (VL) (16 HIV-coinfections) and 69 cutaneous (CL) cases; 85 cases (52 CL) were from the Old World and 20 (17 CL) from the New World. Eight *Leishmania* species were identified, of which 7 were exotic to Italy. VL importation until 1995 was associated with the spread of Mediterranean *Leishmania*-HIV co-infections in early 1990s. Following the introduction of HAART treatment, such cases became occasional in Italians but relatively frequent among immigrants. In contrast, a steady increase of CL cases was observed from different areas of the Old and New Worlds, that in recent years included mainly immigrants ‘visiting friends and relatives’ and Italian tourists. This positive trend likely depends on better diagnosis and reporting; however, we suspect that many CL cases remained unrecognized. Given the relatively low incidence of leishmaniasis importation, the risk of introduction of exotic parasites appears limited, although the detection of anthroponotic species requires attention.

## Introduction

Leishmaniases are a complex of protozoan diseases transmitted by phlebotomine sand flies, with increasing incidence worldwide [1]. About 20 *Leishmania* species are known to infect humans in both the Old and New Worlds, where they cause a variety of clinical conditions broadly grouped in cutaneous (CL), mucosal (ML) and visceral leishmaniasis (VL). Recent estimates of leishmaniasis burden include a global prevalence of 12 million people, with an annual incidence of 0.2–0.4 million VL cases and 0.7–1.2 million CL cases in 101 endemic countries. Over 90% of the VL cases occur in the Indian subcontinent, East Africa and Brazil, whereas elevated CL incidences are reported from several countries of Latin America, southern Mediterranean, Middle East and central Asia [2]. The most widespread entity of leishmaniasis endemic in southern Europe is zoonotic VL, often associated with sporadic CL. Both diseases are caused by *L*.*infantum* and domestic dogs serve as main reservoir hosts. The incidence of clinical disease is relatively low, with an average of about 700 cases reported each year, whereas asymptomatic infections are thought to be widespread [3, 4]. Notification of leishmaniasis has long been compulsory in most southern Europe, including Italy. In non-endemic countries of central and northern Europe, where leishmaniasis is regarded as an imported disease, single or a network of reference centres collect information on a voluntary basis [5].

In the past decade, leishmaniasis—especially CL—has become an international threat for international travelers [5–7]. Traditional (e.g. military personnel) or relatively novel categories of professional travelers (e.g. eco-/adventure tourism workers) are considered at high risk for acquiring the disease. This trend is not only associated with increased travels to endemic destinations, but more and more with labor immigration or refugee populations from endemic zones. In non-endemic countries/regions of Europe, the increase of imported leishmaniasis has been documented by large case series reported from Austria, France (Paris Department), Germany, the Netherlands and United Kingdom [8–20]. VL was mostly acquired in southern Europe, while CL, affecting the majority of cases, was acquired in sub-Saharan and northern Africa, Middle East, central Asia, and the Americas. It should be considered that, in non-endemic areas, imported cases may remain unrecognized owing to the lack of familiarity of physicians with the disease [18]; on the other hand, any diagnosed case of leishmaniasis is recorded as imported. Reports of such cases are comparatively lower in southern Europe [6, 20–22]. While physicians and laboratories from endemic countries can offer greater clinical and diagnostic experience, however they do not always are in the position to distinguish autochthonous from imported cases which might require different care and management.

Monitoring the introduction of exotic parasites in areas at risk of local autochthonous transmission is also important. A possible threat is that, without appropriate surveillance, new *Leishmania* species/genotypes can be introduced and potentially transmitted by local phlebotomine vectors, either parasite specific or permissive ones, with unpredictable consequences [23]. Apart from the historical but emblematic introduction of southern European *L*.*infantum* into the New World [24,25], that suggests parasite plasticity in the adaptation to new eco-systems, the emerge in Europe of new foci sustained by exotic *Leishmania* strains/species, such as *L*.*donovani* in Cyprus and *L*.*major*/*L*.*infantum* hybrids in Portugal [26,27], was recently documented. As a result of environmental modifications, not only vectors, but also mammal hosts may acquire a role in novel transmission cycles [23]. Thus, infected travelers/migrants could also represent potential reservoir hosts for anthroponotic *Leishmania* parasites acquired abroad. Surveillance methods to monitor *Leishmania* introduction by human carriers require both, deep knowledge of global leishmaniasis distribution and adequate parasite identification methods. MultiLocus Enzyme Electrophoresis (MLEE) is still regarded as the reference method for *Leishmania* species identification [1] and, partly thanks to the availability of databases made from thousands isolates, it can also provide fair correlation between enzyme mobility markers and geographic distribution of several parasite populations [28–30]. However MLEE has two main limitations: first, it is laborious and requires parasite cultures, so that only specialized reference centres can afford it; second, in some circumstances MLEE does not seem enough sensitive to discriminate genetic populations from distant geographical locations and having identical biochemical profile, such as members of the widespread zymodeme MON-1 of *L*.*infantum*. Several DNA-based methods have been developed for both species diagnosis and *Leishmania* genotyping at subspecies level (e.g. PCR-RFLPs, Single-Locus Sequencing, MultiLocus Microsatellite Typing-MLMT, and MultiLocus Sequence Typing-MLST) which could have a potential value as geographical marker for imported parasites [25, 31–37]. However the development of a standardized MLST in *Leishmania* taxonomy is relatively recent, so that genotyping information is only available from a limited number of strains within each species and from a limited number of territories [37–40]. As regards MLMT, while it proved to be a powerful approach for tracking parasite populations, its value in assigning a particular geographical origin to individual imported *L*.*infantum* was found limited for our purposes: in fact, a relatively large number of Italian autochthonous *L*.*infantum* isolates were found to share microsatellite variations with populations of this species in the whole Mediterranean area and in Latin America [25].

Since a *Leishmania* Identification Centre was established at Istituto Superiore di Sanità (ISS) for epidemiological purposes [41], the implementation of a surveillance system for imported leishmaniasis in Italy was a priority. This retrospective study reports on the results from 27 years of surveillance based on clinical and epidemiological sources complemented with parasite identification when possible.

## Material and Methods

### Ethics statement

Clinical samples from patients were collected in the frame of routine diagnosis and/or post-treatment follow up, not including additional or unnecessary invasive procedures and after obtaining patient’s informed written consent delivered at the time of clinical examination. Patient records/information was anonymized and de-identified prior to analysis. Data were recorded according to the ISS ethic committee (www.iss.it) that specifically approved this study.

### Patients and epidemiological information

Data on age, sex, nationality, usual residence, medical history, year of diagnosis, clinical features, and tests for leishmaniasis diagnosis were recorded. The putative country/region where *Leishmania* was acquired was inferred by specific queries to patients analyzed along with relevant epidemiological information:

- Because of possible long incubation periods, especially for VL, history of travel(s) to/from endemic areas by both nationals and foreigners was documented for at least 2 years before the disease onset;
- For national and foreigners, the leishmaniasis endemic status of the visited or origin country, respectively, was derived from available scientific literature and World Health Organization guidelines [1];
- The usual residence of national or foreigners patients in the Italian territory was stratified by autochthonous zoonotic infection risk. Traditional human-case notification maps were replaced by the recently available canine leishmaniasis (CanL) seroprevalence prediction map. It identifies most of the Italian territory as belonging to low (estimated seroprevalence <5%), medium-low (5–10%), medium-high (10.01 to 20%), high (20.01–30%) or very high (>30%) endemicity classes [42];
- Type and distribution of *L*. *infantum* zymodemes autochthonous in Italy was established, based on parasite identification data collated at ISS in a 30-year period from over 700 leishmaniasis patients who have indisputably acquired the infection in our country;
- Global distribution maps of *Leishmania* species, zymodemes and genotypes available from scientific literature and unpublished information from WHO *Leishmania* Identification Centres, were used.

Clinical samples for diagnosis or parasite isolates were obtained by hospitals (mainly pediatrics, internal medicine, infectious diseases and dermatology wards) and private dermatologists from throughout the country.

### Samples

In all patients suspected for leishmaniasis, the diagnosis was confirmed by the analysis of different clinical samples depending on the disease form: serum, bone marrow (BM), peripheral blood (buffy-coat, BC) and skin (SK) biopsy. Our laboratory received fresh, frozen and/or fixed samples (slit smears, paraffin sections) sent by hospitals or practitioners. Different assays were used in combination to confirm leishmaniasis diagnosis, as recommended by World Health Organization guidelines [1].

### Diagnosis of disease

#### Serology

In all VL cases and in some CL patients in whom a dissemination of parasites was suspected, the presence of anti-*Leishmania* antibodies was investigated by serology following a long established in-house IFAT protocol using fresh antigen (*L*.*infantum* MHOM/TN/80/IPT1 promastigotes, WHO reference strain of *L*.*infantum*) and FITC-conjugated anti-human immunoglobulins polyvalent serum (Sigma) [43].

#### Parasitology

A combination of classical and molecular parasitological methods were employed: microscopy, *in vitro* culture and polymerase chain reaction (PCR). Microscopic observation was carried out on slide smeared material, mainly BM aspirate for VL and SK punch biopsy or scraping from the edge of the lesion for CL. Fresh material was seeded in Evan’s Modified Tobie’s Medium (EMTM) and ‘Sloppy Evans’ [44,45] and cultures were checked for promastigotes growth up to 30 days. *Leishmania*-positive cultures were cryopreserved pending biochemical and/or molecular characterization. BM, BC and SK fresh or frozen samples were submitted to DNA extraction using the Easy-DNA kit (Invitrogen, San Diego, CA). Specimens fixed on slides were scraped in Tris-EDTA buffer; paraffin sections were de-waxed by using xylene and ethanol at different concentrations before DNA extraction; parasite DNA was extracted by proteinase K [46]. For *Leishmania* sp. detection, genomic DNA was submitted to a nested (n-)PCR assay [47,48]. Three negative controls (BM and SK DNA from healthy patients and no DNA) and one positive control (DNA from promastigotes of MHOM/TN/80/IPT1 strain) were used.

### *Leishmania* identification

#### MultiLocus enzyme electrophoresis

Biochemical typing of cultured *Leishmania* isolates was obtained by MLEE following the zymodeme (Z) Montpellier (MON-) and, in some cases, Rome (ZROM-) nomenclature. *Leishmania* stocks were characterized by starch gel electrophoresis for the analysis of 15 enzymatic systems: phosphoglucomutase (PGM; E.C.2.7.5.1); glucose-phosphate isomerase (GPI; E.C.5.3.1.9); glutamate-oxaloacetate transaminases (GOT1, GOT2; E.C.2.6.1.1.); malic enzyme (ME; E.C.1.1.1.40); phosphogluconate dehydrogenase (6PGD; E.C.1.1.1.44); glucose-6-phosphate dehydrogenase (G6PD; E.C.1.1.1.49); malate dehydrogenase (MDH; E.C.1.1.1.37); nucleoside phosphorylases 1 and 2 (NP1, NP2; E.C.2.4.2.1, E.C.4.2.1.*); mannose-phosphate isomerase (MPI; E.C.5.3.1.8); isocitrate dehydrogenase (ICD; E.C.1.1.1.42); diaphorase NADH (DIA; E.C.1.6.2.2); glutamate-dehydrogenase (GLUD; E.C.1.4.1.3); fumarate hydratase (FH; E.C.4.2.1.2) [49]. WHO reference strains of *L*.*infantum* ZMON-1 (MHOM/TN/80/IPT1), *L*.*donovani* ZMON-2 (MHOM/IN80/DD8), *L*.*major* ZMON-4 (MHOM/SU/73/5-ASKH), *L*.*tropica* ZMON-60 (MHOM/SU/74/K27), and *L*.*aethiopica* ZMON-69 (MHOM/ET/72/L100) for the Old World and *L*.*braziliensis* (MHOM/BR/75/M2903), *L*.*panamensis* (MHOM/PA/71/LS94), *L*.*mexicana* (MHOM/BZ/82/BEL21), and *L*.*guyanensis* (MHOM/BR/75/M4147) for the New World were routinely used. Additional strains were selected as reference for zymodemes representative of the geographical species diversity within the subgenus *Leishmania* (from both the Old and New Worlds) and *Viannia*, from the New World. Results were analyzed for the association of single/multiple isoenzyme markers with geographical regions according to published literature [24,28–30,49,50], and unpublished information collected at our Centre.

#### PCR-RFLP

In clinical samples and cultures, molecular identification of *Leishmania* at species level was achieved by PCR-RFLP analysis of different *Leishmania* target sequences, used alternatively or in combination: a) primers T2 and B4 amplified a nuclear repetitive DNA sequence [51]; b) primers LITSR and L.5.8S amplified the internal transcribed spacer-1 (ITS-1) sequence separating the genes coding for SSU rRNA and 5.8S rRNA [31,52]. *Leishmania* DNA from WHO reference strains reported above were used as controls. Ten μl of PCR products were digested overnight in a total volume of 20 μl, with 10U of *Hae*III restriction enzyme, as recommended by the manufacturer (Promega). All PCR-RFLP products were subjected to electrophoresis by 4% MethaPhor gel (EuroClone) or by Qiaxcel capillary electrophoresis (Qiagen GmbH, Hilden, Germany).

## Results

### Epidemiology

**Fig 1:**
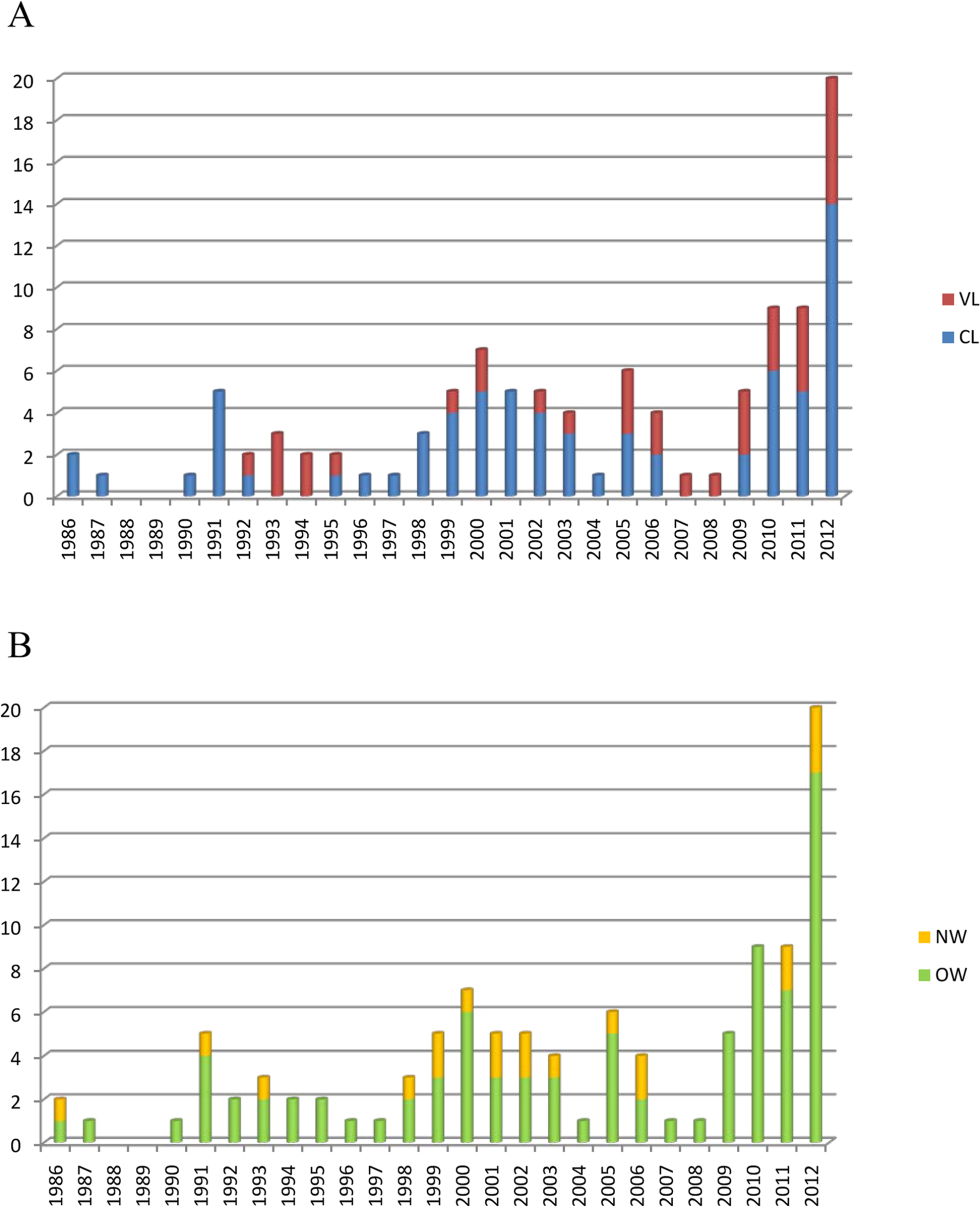
Imported leishmaniasis in Italy, annual trend 1986–2012. A) Distribution of VL and CL cases. B) Distribution of Old and New World cases.

Leishmaniasis imported in Italy has been systematically documented at ISS since 1986. Thirty-four National Health System or University hospitals and private dermatologists from 16/20 Italian Regions referred to us patients with a suspect of imported disease. One hundred five patients with laboratory-confirmed leishmaniasis cases were recorded as suspected of importation through 2012. Of the them, 36 were diagnosed as having VL and 69 CL. A total of 83 *Leishmania* strains were cultured and cryopreserved from 80 patients. The disease was recorded in all age groups (range: 2–74 years) and in both sexes, but it was more frequently diagnosed in adults (88 patients, 83.8%) and in males (81.9%) (S1 Table). The annual trend of cases presented fluctuations with an evident increase after 2009; each year, the patients ranged from nil in 1989 to a maximum of 20 cases recorded in 2012 (Fig 1A).

**Fig 2:**
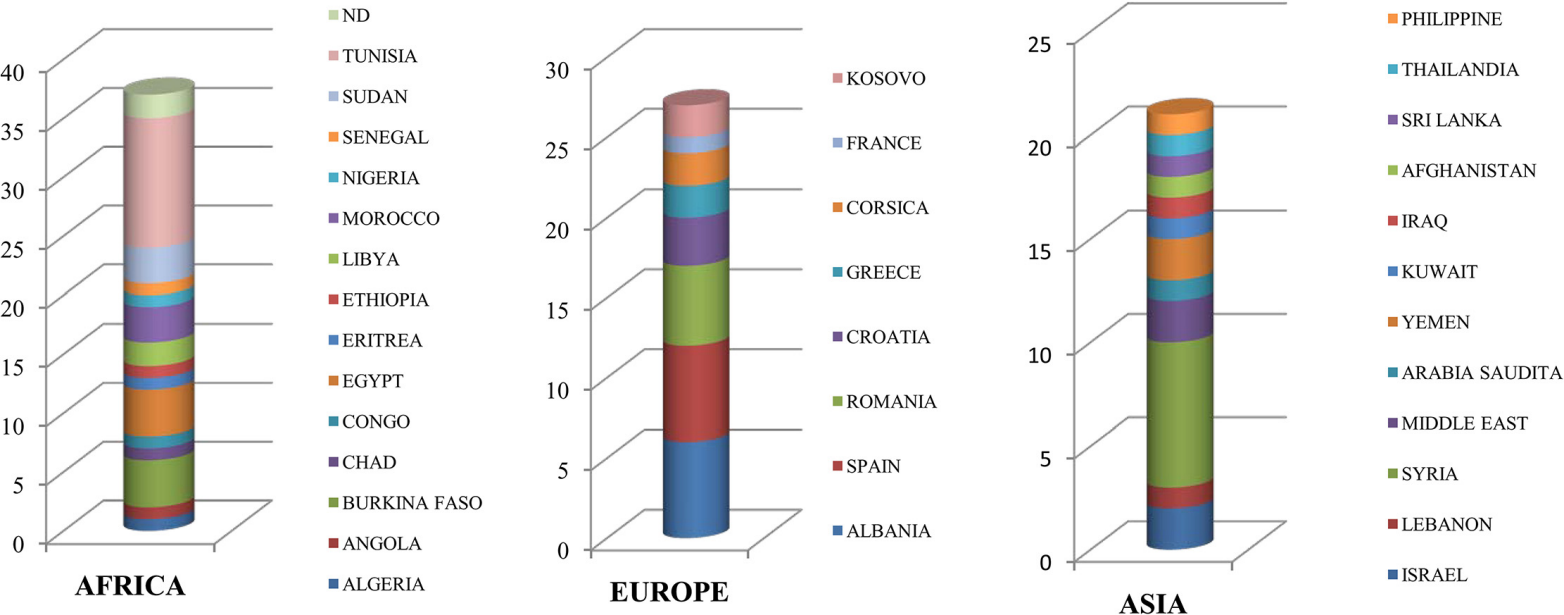
Number of leishmaniasis cases imported from Old World countries.

The analysis of the epidemiological records in combination with *Leishmania* identification indicated that the geographical origin of infection was the Old World in 85 cases (52 CL), and the New World in 20 cases (17 CL) (Fig 1A and 1B). Old World cases were distributed as follows: 35 cases were from Northern Mediterranean Africa (Algeria, Egypt, Libya, Tunisia and Morocco) and Sub-Saharan Africa (Angola, Burkina Faso, Chad, Congo, Eritrea, Ethiopia, Nigeria, Senegal and Sudan); 2 cases from the African continent without specified country (Fig 2A); 27 cases were from western and eastern south Europe (Albania, Corsica, Croatia, southern continental France, Greece, Kosovo, Romania and Spain) (Fig 2B); 21 cases were from Asia, of which 17 from the Middle East and Arabian Peninsula (Iraq, Israel, Lebanon, Jordan, Kuwait, Saudi Arabia, Syria and Yemen) and 1 case each from Afghanistan, Sri Lanka, Thailand and Philippines (Fig 2C). As regards cases from the New World, 7 were from North (Mexico) and Central America (Costa Rica and Guatemala), and 13 from South America (Bolivia, Brazil, Colombia, Ecuador, Peru and Venezuela).

As shown in Table 1, the typology of patients changed over time. Italian tourists (47.6%) and immigrants (45.7%) were recorded at similar rates; the latter included both recent immigrants (19.0%) and naturalized immigrants visiting friends and relatives (VFR, 26.7%). VFR cases, however, were mainly recorded from 2009. Rare cases occurred in military staff during the second Gulf War in Kuwait and Iraq (n = 2), in missionaries in Chad, Ethiopia, Burkina Faso and Spain (n = 4), and in an adopted child from Brazil (S1 Table).

**Table 1:** Typology of travelers detected during the study period (1986–2012).

Type of traveler	1986–1988	1989–1991	1992–1994	1995–1997	1998–2000	2001–2003	2004–2006	2007–2009	2010–2012	Total (%)
**Tourist**	3	4	5	4	11	7	6	-	10	50 (47.6)
**Recent immigrant**	-	2	1	-	2	3	3	5	4	20 (19.0)
**VFR**	-	-	-	-	1	2	1	2	22	28 (26.7)
**Soldier**	-	-	-	-	-	1	1	-	-	2 (1.9)
**Missionary**	-	-	1	-	-	1	-	-	2	4 (3.8)
**Adopted child**	-	-	-	-	1	-	-	-	-	1 (1.0)
**Total**	3	6	7	4	15	14	11	7	38	105

As regards associated conditions, 21 patients (20%) were immunosuppressed (causes being HIV, lymphoma or organ transplantation). Among 16 HIV co-infected cases, 7 consisted of VL patients diagnosed among Italian tourists during 1993–2000, whereas 9 cases detected after 2000 (6 VL and 3 CL) were mainly immigrants. Finally, 12 out of 17 children were immigrants, 6 of whom recorded as VFR after 2010, from countries of the principal migratory fluxes to Italy (Albania, Tunisia and Syria) (S1 Table).

### *Leishmania* identification

Initially, *Leishmania* isolates were typed by MLEE alone; afterwards, MLEE and/or PCR-RFLP methods were used. When a *Leishmania* culture was not available, parasites were identified at species level by PCR-RFLP applied on clinical samples.

Agent identification was achieved in 84/105 patients. Eight *Leishmania* species were detected: *L*.*infantum* in 33 patients affected by VL or CL, *L*.*major* in 22 (CL), *L*.*tropica* in 10 (CL), *L*.*braziliensis* in 8 (CL), *L*.*panamensis* in 5 (CL), *L*.*mexicana* in 2 (CL), *L*.*aethiopica* (CL) and *L*.*donovani* (VL) in one patient each. Furthermore, parasites belonging to *L*.*donovani* complex (VL) and *L*.*guyanensis* complex (CL) were identified in one patient each. Clinical samples were found inadequate for typing in the remaining 21 cases (8 VL, and 13 CL) (Table 2).

**Table 2:** *Leishmania* species identified in imported leishmaniasis cases according to area of infection and travel type.

*Leishmania spp*.	No. cases	Clinical form	Origin of infection Area (countries)	Traveler type
*L*.*infantum*	26	VL	Southern Europe (Albania, Corsica, France, Greece, Spain, ex Yugoslavia); Africa (Egypt, Angola^a^, Congo^b^, Nigeria^a^); South America (Brazil)	Turists, Immigrants
	7	CL		
*L*.*major*	22	CL	Northern (Libya, Morocco, Tunisia) and Sub-SaharanAfrica (Sudan, Chad, Burkina Faso); Middle East (Syria, Yemen, Iraq)	Turists, Immigrants, VFRs, Missionary, Soldiers
*L*.*tropica*	10	CL	Middle East (Israel, Syria, Jordan); Northern Africa (Tunisia, Morocco)	Turists, Immigrants
*L*.*aethiopica*	1	CL	Africa (Ethiopia)	Missionary
*L*.*donovani* complex	1	VL	Africa (Eritrea^b^)	Immigrant
*L*.*donovani*	1	VL	Asia (Philippines)	VFR
*L*.*panamensis*	5	CL	North (Mexico) and Central America (Costa Rica)	Tourists
*L*.*guyanensis* complex	1	CL	South America (Bolivia)	Tourist
*L*.*mexicana*	2	CL	North (Mexico) and Central America (Guatemala)	Tourist
*L*.*braziliensis*	8	CL	North (Mexico) and South America (Brazil, Peru, Bolivia, Colombia, Ecuador)	Tourists, Adopted child
unidentified	9	VL	Southern and Eastern Europe (Spain, Croatia, Albania, Kosovo Romania); Middle East (Syria); South East Asia (Sri Lanka and Thailand); South America (Brazil and Ecuador)	Tourists, Missionary, Immigrants, VFRs
	12	CL		

VL, Visceral leishmaniasis; CL, Cutaneous leishmaniasis.

^a^countries where VL is not declared [1]

^b^countries where VL and CL are recorded but the *Leishmania* species was not identified [1].

MLEE was performed on 52 isolates, of which 40 were identified as Old World and 12 as New World *Leishmania* species. Zymodeme profiles contributed to clarify the geographical origin of the infection in several cases (Table 3 and S1 Table). Out of 17 strains typed as *L*.*infantum*, 8 zymodemes were detected. Nine strains belonged to ZMON-1, the commonest zymodeme causing zoonotic VL worldwide; patients originated from, or reported travels to Albania, Greece, Croatia, Spain, France, Angola and Brazil [28,49,53–55]. Five dermotropic zymodemes were detected in HIV-co-infected patients with VL: ZMON-11 (2 cases), ZMON-29, ZMON-190 and ZMON-228 (1 patient each), reported from Spain and/or France [28,30,56–58], and ZMON-189 from Croatia. Finally, 2 uncommon zymodemes ZMON-34 and ZMON-105, known to be spread over the Mediterranean area [49,58–60], were isolated from patients who acquired the infection in Corsica. We identified four zymodemes of *L*.*major* from CL patients: ZMON-25, a widespread zymodeme recorded from Libya, Morocco and Tunisia [29,49]; ZMON-4 from Sudan and Saudi Arabia [49]; ZMON-26 from Yemen, Syria and Burkina Faso and ZMON-196 from Chad [29]. Four zymodemes of *L*.*tropica*, including three new zymodemes (S2 Table), were also identified as agents of CL: ZMON-102 (1 patient), a zymodeme recorded from Morocco [29]; ZROM-96 (1 patient) and ZROM-107 (2 patients) having enzyme profile markers in common with zymodemes reported from Middle East (Jordan, Syria and Palestine) and Tunisia [29,61,62]; and ZROM-93 (1 patient) similar to zymodemes recorded from Jordan, Israel, Palestine and Syria [62,63]. A new zymodeme of *L*.*aethiopica*, ZROM-106, was identified in a CL patient from Ethiopia (S2 Table).

**Table 3:** *Leishmania* zymodemes identification by MLEE and related geographic origin of infection.

*Leishmania spp*	Immunological status	Clinical form	Zymodeme (No. strains)	Geographical origin of infection	References
*L*.*infantum*	HIV+	VL	MON-189	Croatia	(28)
	HIV+	VL	MON-190	Spain	(28)
	HIV+	VL	MON-11 (2)	France, Spain	(28,58)
	HIV+	VL	MON-29	Spain	(28,56,57)
	HIV+	VL	MON-228	Spain	(28)
	IC	CL	MON-34	Corsica	(unpublished)
	IC	VL	MON-105	Corsica	(unpublished)
	HIV+	VL	MON-1	Brazil	(28,49)
	HIV+	VL	MON-1	Spain	(28,49)
	IC	VL	MON-1 (2)	Greece, Croatia	(28,49)
	IC	CL	MON-1	Greece	(53)
	I, IC	VL	MON-1(2)	Albania	(55)
	HIV+	VL	MON-1	France	(49)
	I	VL	MON-1	Angola	(54)
*L*.*major*	IC	CL	MON-25 (8)	Morocco, Libya, Tunisia	(29,49)
	IC	CL	MON-26 (4)	Syria, Yemen, Burkina Faso	(29)
	IC	CL	MON-196	Chad	(29)
	IC	CL	MON-4 (4)	Sudan, Saudi Arabia	(49)
*L*.*tropica*	IC	CL	ROM-93	Israel, Syria	(unpublished)
	IC	CL	ROM-96	Israel, Syria, Jordan	(unpublished)
	IC	CL	ROM-107(2)	Tunisia, Israel Syria, Jordan	(unpublished)
	IC	CL	MON-102	Morocco	(29)
*L*.*aethiopica*	IC	CL	ROM-106	Ethiopia	(unpublished)

I, immunocompromised; IC, immunocompetent; MON-, Montpellier nomenclature zymodemes; ROM-, Rome nomenclature zymodemes.

### Case definition and classification

**Fig 3:**
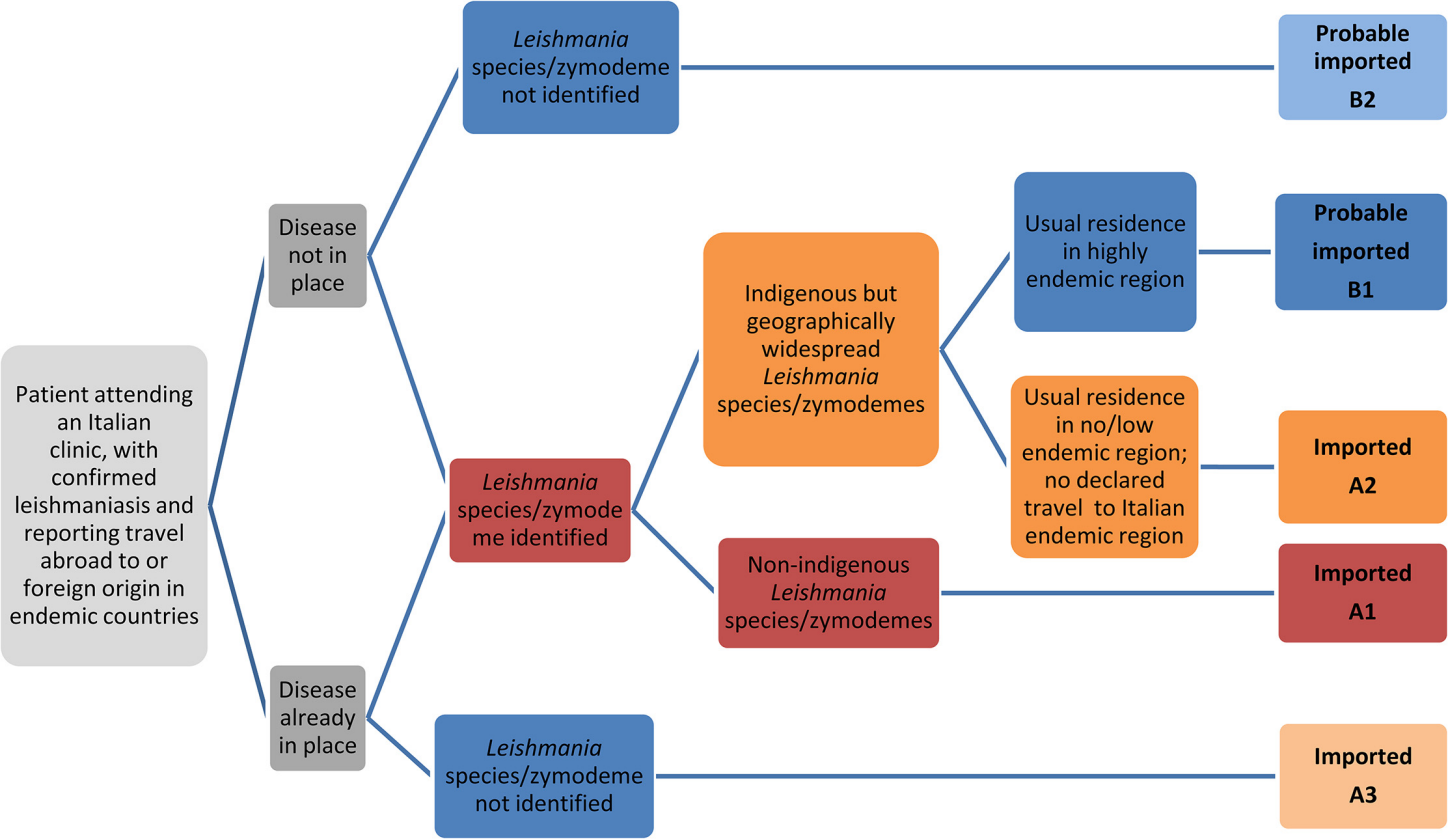
Algorithm used for the classification of suspected cases of imported leishmaniasis in “imported” (certainty degrees A1, A2, A3) and “probably imported” (certainty degrees B1, B2).

The algorithm shown in Fig 3 was designed to combine epidemiological and parasite bio-molecular information and it was used to classify leishmaniasis cases as “imported” (group A; 84 cases) or “probably imported” (group B; 21 cases) because of various degrees of evidence. Sub-group A1, representing indisputable imported disease, consisted of 56 patients infected by exotic *Leishmania* species or by *L*.*infantum* zymodemes non-autochthonous in Italy (ZMON-11, ZMON-29, ZMON-105, ZMON-190 and ZMON-228) (S1 Table). Sub-group A2 included 14 patients for whom parasite identification gave non-discriminating results, i.e. the agents were identified as *L*.*infantum* zymodemes autochthonous in Italy but also elsewhere (ZMON-1 and ZMON-189), or as *L*.*infantum* species without further genotype information as resulting by PCR-RFLP identification. However there was still strong indication for leishmaniasis importation inferred by travel history, i.e. the disease was already in place upon arrival to Italy, and/or the patient’s residence in Italy was in a non-endemic territory with no declared travels to endemic Italian regions. Sub-group A3 consisted of 14 patients for whom parasite identification was unavailable but they showed good indication for importation based on history, i.e. the disease was already in place or developed shortly after the arrival in Italy, or the month of arrival was incompatible with the autochthonous transmission period. The B group consisted of patients for whom there was still strong indication for leishmaniasis importation inferred by patient’s travel history, however parasite identification gave non-discriminating results (B1) or was unavailable (B2). Sub-group B1 included 14 patients infected by widespread *L*.*infantum* zymodemes (ZMON-1 and ZMON-34), or by “generic” *L*.*donovani* complex or *L*.*infantum* species as determined by PCR-RFLP. Sub-group B2 consisted of 7 patients with suggestive travel history but *Leishmania* typing was unavailable. Regarding this group, a VL case was an immigrate from Nigeria (Table 2), a country where the disease is not reported as endemic [1], however we cannot rule out the possibility of travels across endemic African countries before the arrival in Italy.

## Discussion

Currently, leishmaniasis is considered a dynamic disease: different causes such as global warming (affecting the ecology and distribution of the phlebotomine vectors) and man-made risk factors (travels, migration, trade of animals and environmental modifications) are changing the temporal-spatial evolution of leishmaniasis in Europe [21, 23]. In relation to this, the introduction of exotic parasites can also be a matter of concern. We believe that in the current eco-epidemiological situation and thanks to the fair development of health care systems, *Leishmania* epidemics could hardly involve large numbers of individuals with high fatality rates, whatever *Leishmania* species is introduced. Rather, the main risk would be the insidious introduction (because unrecognized) of an agent that can adapt to local reservoirs and vectors. Therefore our surveillance is not limited to imported human parasites, but it extends to infections of domestic and wild reservoirs, and to monitoring geographical diffusion and seasonal dynamics of autochthonous phlebotomine vectors [7,64].

The purpose of our study was to provide the magnitude and diversity of imported leishmaniasis over 27 years of surveillance. This was based, although not exclusively, on parasite identification. When this was impossible, other important information was considered in the analysis, that included clinical (e.g. incubation period, skin lesions appearance, etc) and epidemiological data (relative risk for visited/origin versus residence places). Our methods of *Leishmania* species identification changed over time, having become more sensitive and rapid using reproducible and internationally validated PCR-RFLPs on clinical samples [31,51]. Identification results have been used to support the above algorithm, whereas we did not intend to investigate on the molecular phylogeny of a given *Leishmania* population starting from a single imported parasite. To overcome MLEE limitations in discriminating geographical genotypes within largely diffuse *L*.*infantum* zymodemes, MLST appears potentially the most powerful approach and will, most probably, replace MLEE in the future. However biases due to small sample sizes and the absence of inter-laboratory standardization should be avoided. To this end, accessible sequence databases should be created and sustained for integrating data obtained by different researchers [37].

Our findings indicated a relatively low incidence of leishmaniasis importation into Italy during the 1986–2012 period (n = 105, for an average of 4 cases/year). These cases represented approximately 3.5% of all leishmaniasis cases notified in Italy (n = 3028), and 6.6% of those diagnosed at ISS (n = 1596), during the same period [64]. However our results may suffer from underestimation. First, the performance of VL notification system in Italy, although acceptable, varies from region to region, whereas CL is definitely underreported at national level because frequently it does not require hospitalization; furthermore, notification records of both diseases do not include specific information on possible disease importation. Second, several health centres specialized in tropical diseases are available at regional level, consequently leishmaniasis diagnosis is not centralized. Hence, our surveillance could be biased by preferential contacts of some peripheral units with our reference centre at ISS. Low reporting of imported leishmaniasis seems to occur in other endemic countries of southern Europe; in Greece, only 2.8% of CL cases from 1998 to 2011 were classified with a ‘possibility of being imported’ [65]; similarly, very few patients were routinely recorded as such in Spain [22]. In contrast, high incidences of imported leishmaniasis are recorded in non-endemic northern and central Europe, with a range by country of 20 to 82.5 cases/year [10–21,66–68]. It may be due to different reasons: i) leishmaniasis diagnosis is often centralized at dedicated centres which, in addition, raise awareness and disseminate information among physicians and travelers; ii) some countries have consistent military contingents in endemic areas for training and active duty and, accordingly, an increase of leishmaniasis importation is clustered in periods coinciding with specific conflicts; iii) there is a relevant number of migrants and VFRs from old colonies steadily importing tropical diseases.

Nevertheless we have observed an increasing trend in Italy, that may be associated with an actual increase of *Leishmania* importation but also with better awareness among clinicians and improvement of diagnostic tools. More practical *Leishmania* typing techniques have replaced the laborious and centralized MLEE, often enabling the retrospective identification of agents from archived samples. Our case series included prevalently adult males (69.5%) and CL was the most frequent form (65.7%), being steadily imported throughout the study period from various Old and New World areas. An increase of tegumentary forms was diagnosed in novel typologies of Italian eco-tourists and adventure travelers who were infected in the Amazon region. Changes in the patient’s characteristics are noteworthy as regards the associated HIV condition. During the 1990s, imported VL infections by Mediterranean *L*.*infantum* zymodemes were frequently diagnosed among Italian HIV-positive tourists [28]. Following the introduction of HAART treatment in Europe in late 1990s, such cases became occasional whereas co-infections was more often diagnosed in immigrants, as a probable result of limited access to antiretroviral therapies.

As regards Old World agents, the geo-localization of imported *L*.*infantum*, *L*.*tropica*, *L*.*major* and *L*.*aethiopica* as resulting from MLEE analysis was fully consistent with travel history in most patients. For other cases the attribution of ‘imported’ versus ‘autochthonous’ was more problematic. This occurred mainly with the immigrant population, for which travel history was often incomplete as regards travels performed before entry or during the stay in Italy. Our algorithm reflects such situation: recent immigrants and VFR represent a low percentage of evident “imported” cases (group A: 31/84, 36.9%), whereas they are the majority of those “probably imported” (group B: 17/21, 80.9%). It should be pointed out that we routinely detect cases in immigrants who have certainly acquired VL in Italy, either because found infected with a *L*. *infantum* zymodeme (MON-72) exclusively endemic in the Campania region of Italy [64], or because they spent more than 2 years in highly endemic territories of the country.

In conclusion, despite our data are likely to be underestimated, they evidenced the introduction of a broad spectrum of parasites representative of 8 human species, of which 7 exotic to Italy. Given the low incidence of leishmaniasis importation, the risk of permanent introduction of exotic species/genotypes appears limited. However such risk should be diversified; zoonotic species such as *L*. *major* or neotropical *Leishmania* could unlikely be introduced in Italy because lacking of natural reservoir hosts; on the contrary, a risk associated with the importation of anthroponotic species (*L*. *tropica* and *L*. *donovani*) may require attention in the event of increased frequency [23,27,69]. This emphasizes the importance of monitoring imported leishmaniasis prospectively so that changes in patterns and emerging risk factors can be identified.

## Supporting Information

S1 Table Supporting cumulative information of imported leishmaniasis cases. Abbreviations: VL, Visceral leishmaniasis; CL, Cutaneous leishmaniasis; A, adult cases: >17 years old; P, pediatric cases: 0–16 years old; M, male; F, female; ND, not done; NR, not reported; MON-, Montpellier nomenclature; ROM-, Rome nomenclature; A1, A2, A3, certainty degrees of “imported cases”; B1, B2, certainty degrees of “probable imported cases”.
(XLS)

S2 Table Zymodeme profile of four ZROM- zymodemes. Abbreviations of 15 iso-enzymatic sistems: malate dehydrogenase, MDH, EC 1.1.1.37; malic enzyme, ME, EC 1.1.1.40; isocitrate dehydrogenase, ICD, EC 1.1.1.42; 6-phosphogluconate dehydrogenase, PGD, EC 1.1.1.44; glucose-6-phosphate dehydrogenase, G6PD, EC 1.1.1.49; glutamate dehydrogenase, GLUD, EC 1.4.1.3; NADH diaphorase, DIA, EC 1.6.2.2; purine nucleoside phosphorylase, NP1, EC 2.4.2.1; purine nucleoside phosphorylase, NP2, EC 2.4.2.*; glutamate-oxaloacetate transaminases, GOT1 and GOT2, EC 2.6.1.1; phosphoglucomutase, PGM, EC 5.4.2.2; fumarate hydratase, FH, EC 4.2.1.2; mannose phosphate isomerase, MPI, EC 5.3.1.8; glucose phosphate isomerase, GPI, EC5.3.1.9.
(XLS)

## References

[1] World Health Organization (WHO) Control of the leishmaniases, Geneva, WHO, 2010.

[2] Alvar J, Velez ID, Bern C, Herrero M, Desjeux P, Cano J et al The WHO Leishmaniasis Control Team. Leishmaniasis Worldwide and Global Estimates of Its Incidence. PLoS ONE. 2012;7(5): 35671. 10.1371/journal.pone.0035671 PMC3365071 22693548

[3] Michel G, Pomares C, Ferrua B, Marty P. Importance of worldwide asymptomatic carriers of *Leishmania infantum* (*L*. *chagasi*) in human. Acta Trop. 2011;119: 69–75. 10.1016/j.actatropica.2011.05.012 21679680

[4] Šiško-Kraljević K, Jerončić A, Mohar B, Punda-Polić V. Asymptomatic Leishmania infantum infections in humans living in endemic and non-endemic areas of Croatia, 2007 to 2009. Euro Surveill. 2013;18(29): 20533 23929119

[5] Gradoni L. Epidemiological surveillance of leishmaniasis in the European Union: operational and research challenges. Euro Surveill. 2013;18(30): 20539 23929176 10.2807/1560-7917.es2013.18.30.20539

[6] Antinori S, Gianelli E, Calattini S, Longhi E, Gramiccia M, Corbellino M. Cutaneous leishmaniasis: an increasing threat for travelers. Clinical Microbiology and Infection. 2005;11(5): 343–346. 15819858 10.1111/j.1469-0691.2004.01046.x

[7] Gramiccia M, Gradoni L. The leishmaniases in Southern Europe In: Takken W. & Knols Bart GJ Editors. Emerging pests and Vector- Borne Diseases, Ecology and control of vector-borne diseases, Wageningen Academic Publishers, 2007 pp. 75–95.

[8] Pavli A, Maltezou HC. Leishmaniasis, an emerging infection in travelers. Int J Infect Dis. 2010;14: 1032–1039. 10.1016/j.ijid.2010.06.019 20952234

[9] Harms G, Schönian G, Feldmeier H. Leishmaniasis in Germany. Emerg Infect Dis. 2003; 9(7): 872–875. 12890332 10.3201/eid0907.030023 PMC3023440

[10] El Hajj L, Thellier M, Carrière J, Bricaire F, Danis M, Caumes E. Localized cutaneous leishmaniasis imported into Paris: a review of 39 cases. Int J Dermatol. 2004; 43: 120–125. 15125502 10.1111/j.1365-4632.2004.01991.x

[11] Lawn SD, Whetham J, Chiodini PL, Kanagalingam J, Watson J, Behrens RH, et al New world mucosal and cutaneous leishmaniasis: an emerging health problem among British travelers. Q J Med. 2004;97: 781–788. 10.1093/qjmed/hch127 15569809

[12] Weitzel T, Mühlberger N, Jelinek T, Schunk M, Ehrhardt S, Bogdan C et al Surveillance Importierter Infektionen in Deutschland (SIMPID) Surveillance Network. Imported leishmaniasis in Germany 2001–2004: data of the SIMPID surveillance network. Eur J Clin Microbiol Infect Dis. 2005;24: 471–476. 15997368 10.1007/s10096-005-1363-1

[13] Malik AN, John L, Bryceson AD, Lockwood DN. Changing pattern of visceral leishmaniasis, United Kingdom, 1985–2004. Emerg Infect Dis. 2006;12: 1257–1259. 16965709 10.3201/eid1208.050486 PMC3291201

[14] Herremans T, Pinelli E, Casparie M, Nozari N, Roelfsema J, Kortbeek L. Increase of imported Leishmaniasis in the Netherlands: a twelve year overview (1996–2007). Int Health. 2010;2: 42–46. 10.1016/j.inhe.2009.12.005 24037049

[15] Harms G, Scherbaum H, Reiter-Owona I, Stich A, Richter J. Treatment of imported New World cutaneous leishmaniasis in Germany. Intl J Dermat. 2011;50: 1336–1342. 10.1111/j.1365-4632.2011.04987.x 22004484

[16] Herbinger KH, Siess C, Nothdurft HD, von Sonnenburg F, Löscher T. Skin disorders among travellers returning from tropical and non-tropical countries consulting a travel medicine clinic. Trop Med Int Health. 2011;16: 1457–1464. 10.1111/j.1365-3156.2011.02840.x 21767336

[17] Wall EC, Watson J, Armstrong M, Chiodini PL, Lockwood DN. Short Report: Epidemiology of Imported Cutaneous Leishmaniasis at the Hospital for Tropical Diseases, London, United Kingdom: Use of Polymerase Chain Reaction to Identify the Species. Am J Trop Med Hyg. 2012;86(1): 115–118. 10.4269/ajtmh.2012.10-0558 22232460 PMC3247118

[18] Bart A, van Thiel PP, de Vries HJC, Hodiamont CJ, Van Gool T. Imported leishmaniasis in the Netherlands from 2005 to 2012: epidemiology, diagnostic techniques and sequence-based species typing from 195 patients. Euro Surveill. 2013;18(30): 14–21. 10.2807/1560-7917.es2013.18.30.20544 23929178

[19] Poeppl W, Oeser C, Grabmeier-Pfistershammer K, Walochnik J, Burgmann H. Clinical findings and management of imported cutaneous leishmaniasis: report of 14 cases from Austria. Travel Med Infect Dis. 2013;11: 90–94. 10.1016/j.tmaid.2013.03.002 23522841

[20] Morizot G, Delgiudice P, Caumes E, Laffitte E, Marty P, Dupuy A et al Healing of Old World cutaneous Leishmaniasis in travelers treated with fluconazole: drug effect or spontaneous evolution? Am J Trop Med Hyg. 2007; 76(1): 48–52. 17255228

[21] Dujardin JC, Campino L, Cañavate C, Dedet J-P, Gradoni L, Soteriadou K et al Spread of vector-borne diseases and neglect of Leishmaniasis, Europe. Emerg Infect Dis. 2008;14: 1013–1018. 10.3201/eid1407.071589 18598618 PMC2600355

[22] Pérez-Ayala A, Norman F, Pérez-Molina JA, Herrero JM, Monge B, Lopez-Velez R. Imported Leishmaniasis: A Heterogeneous Group of Diseases. J Trav Med. 2009;16(6): 395–401. 10.1111/j.1708-8305.2009.00341.x 19930379

[23] Antoniou M, Gramiccia M, Molina R, Dvorak V, Volf P. The role of indigenous phlebotomine sandflies and mammals in the spreading of leishmaniasis agents in the Mediterranean region. Euro Surveill. 2013;18(30): 54–61. 10.2807/1560-7917.es2013.18.30.20540 23929183

[24] Rotureau B, Ravel C, Aznar C, Carme B, Dedet JP. First report of *Leishmania infantum* in French Guiana: canine visceral leishmaniasis imported from the Old World. J Clin Microbiol. 2006; 44: 1120–1122. 16517909 10.1128/JCM.44.3.1120-1122.2006 PMC1393124

[25] Kuhls K, Alam MZ, Cupolillo E, Ferreira GE, Mauricio IL, Oddone R et al Comparative microsatellite typing of new world *Leishmania infantum* reveals low heterogeneity among populations and its recent old world origin. PLoS Negl Trop Dis. 2011;5(6): 1155. 10.1371/journal.pntd.0001155 PMC3110170 21666787

[26] Antoniou M, Haralambous C, Mazeris A, Pratlong F, Dedet J-P, Soteriadou K. *Leishmania donovani* leishmaniasis in Cyprus. Lancet Infect Dis. 2008; 8: 6–7. 18156082 10.1016/S1473-3099(07)70297-9

[27] Ravel C, Cortes S, Pratlong F, Morio F, Dedet JP, Campino L. First report of genetic hybrids between two very divergent Leishmania species: *Leishmania infantum* and *Leishmania major* . Int J Parasitol. 2006;36: 1383–1388. 16930606 10.1016/j.ijpara.2006.06.019

[28] Gramiccia M. The identification and variability of the parasites causing leishmaniasis in HIV-positive patients in Italy. Ann Trop Med Parasitol. 2003;97(S1): 65–73. 14678634 10.1179/000349803225002543

[29] Pratlong F, Dereure J, Ravel C, Lami P, Balard Y, Serres G et al Geographical distribution and epidemiological features of Old World cutaneous leishmaniasis foci, based on the isoenzyme analysis of 1048 strains. Trop Med Int Health. 2009;14(9): 1071–1085. 10.1111/j.1365-3156.2009.02336.x 19624480

[30] Pratlong F, Lami P, Ravel C, Balard Y, Dereure J, Serres G et al Geographical distribution and epidemiological features of Old World Leishmania infantum and Leishmania donovani foci, based on the isoenzyme analysis of 2277 strains. Parasitol. 2013;140(4): 423–434. 10.1017/S0031182012001825 23146283

[31] Schönian G, Nasereddin A, Dinse N, Schweynoch C, Schallig HD, Presber W et al PCR diagnosis and characterization of Leishmania in local and imported clinical samples. Diagn Microbiol Infect Dis. 2003;77(1): 349–358. 10.1016/s0732-8893(03)00093-2 12967749

[32] Marfurt J, Niederweiser I, Makia ND, Beck HP, Felger I. Diagnostic genotyping of old and new world Leishmania species by PCR–RFLP. Diagn Microbiol Infect Dis. 2003;46: 115–124. 12812715 10.1016/s0732-8893(03)00040-3

[33] Kuhls K, Mauricio IL, Pratlong F, Presber W, Schönian G. Analysis of ribosomal DNA internal transcribed spacer sequences of the Leishmania donovani complex. Microbes Infect. 2005;7: 1224–1234. 16002315 10.1016/j.micinf.2005.04.009

[34] Kuhls K, Chicharro C, Canavate C, Cortes S, Campino L, Haralambous C et al Differentiation and Gene Flow among European Populations of *Leishmania infantum* MON-1. PLoS Negl Trop Dis. 2008;2(7): e261 10.1371/journal.pntd.0000261 18612461 PMC2438616

[35] Alam MZ, Haralambous C, Kuhls K, Gouzelou E, Sgouras D, Soteriadou K et al The paraphyletic composition of Leishmania donovani zymodeme MON-37 revealed by multilocus microsatellite typing. Microbes Infect. 2009;11: 707–15. 10.1016/j.micinf.2009.04.009 19376262

[36] Botilde Y, Laurent T, Quispe Tintaya W, Chicharro C, Canavate C, Cruz I et al Comparison of molecular markers for strain typing of *Leishmania infantum* . Inf Gen Evol. 2006;6: 440–446. 10.1016/j.meegid.2006.02.003 16581311

[37] Van der Auwera G, Ravel C, Verweij JJ, Bart A, Schönian G, Felger I. Evaluation of Four Single-Locus Markers for Leishmania Species Discrimination by Sequencing. J Clin Microbiol. 2014;52(4): 1098–1104. 10.1128/JCM.02936-13 24452158 PMC3993476

[38] Boité MC, Mauricio IL, Miles MA, Cupolillo E. New insights on taxonomy, phylogeny and population genetics of Leishmania (Viannia) parasites based on multilocus sequence analysis. PLoS Negl Trop Dis. 2012;6(11): e1888 10.1371/journal.pntd.0001888 23133690 PMC3486886

[39] El Baidouri F, Diancourt L, Berry V, Chevenet F, Pratlong F et al Genetic structure and evolution of the Leishmania genus in Africa and Eurasia: what does MLSA tell us. PLoS Negl Trop Dis. 2013;13:7: e2255 10.1371/journal.pntd.0002255 23785530 PMC3681676

[40] Chicharro C, Llanes-Acevedo IP, García E, Nieto J, Moreno J, Cruz I. Molecular typing of Leishmania infantum isolates from a leishmaniasis outbreak in Madrid, Spain, 2009 to 2012. Euro Surveill. 2013;8(30): 20545 23929179 10.2807/1560-7917.es2013.18.30.20545

[41] World Health Organization (WHO) Control of the leishmaniases. Geneva 1990. Tech Rep Ser; p793.

[42] Franco AO, Davies CR, Mylne A, Dedet J-P, Gállego M, Ballart C et al Predicting the distribution of canine leishmaniasis in Western Europe based on environmental variables. Parasitol. 2011;138: 1878–1891. 10.1017/S003118201100148X 21914251

[43] Gradoni L, Scalone A, Gramiccia M. HIV-Leishmania co-infections in Italy: serological data as an indication of the sequence of acquisition of the two infections. Trans R Soc Trop Med Hyg. 1993;87: 94–96. 8465412 10.1016/0035-9203(93)90441-r

[44] Evans DA. Leishmania In: Taylor A.E.R. and Baker J.R. editors. In vitro methods for parasites cultivation., Academic Press, London and New York 1987 pp 58–59.

[45] Gramiccia M, Gradoni L. Successful in vitro isolation and cultivation of Italian dermotropic strains of *Leishmania infantum* sensu lato. Trans R Soc Trop Med Hyg. 1989;83(1): 76 2603211 10.1016/0035-9203(89)90713-x

[46] Khatri M, Di Muccio T, Gramiccia M. Cutaneous leishmaniasis in North-Western Yemen: A clinicoepidemiologic study and Leishmania species identification by polymerase chain reactionerestriction fragment length polymorphism analysis. J Am Acad Dermatol. 2008;61(4): 15–21. 10.1016/j.jaad.2009.04.047 19695737

[47] Van Eys GJ, Schoone GJ, Kroon NC, Ebeling SB. Sequence analysis of small subunit ribosomal RNA genes and its use for detection and identification of Leishmania parasites. Mol Biochem Parasitol. 1992;51: 133–142. 1565128 10.1016/0166-6851(92)90208-2

[48] Cruz I, Cañavate C, Rubio JM, Morales MA, Chicharro C, Laguna F et al Spanish HIV-Leishmania Study Group. A nested polymerase chain reaction (Ln-PCR) for diagnosing and monitoring Leishmania infantum infection in patients co-infected with human immunodeficiency virus. Trans R Soc Trop Med Hyg. 2002;96(S1): 185–189. 12055836 10.1016/s0035-9203(02)90074-x

[49] Rioux JA, Lanotte G, Serres E, Pratlong F, Bastien P, Perieres J. Taxonomy of Leishmania. Use of isoenzymes. Suggestions for a new classification. Ann Parasitol Hum Comp. 1990; 65: 11–125. 2080829 10.1051/parasite/1990653111

[50] Cupolillo E, Grimaldi G Jr, Momen H. A general classification of New World Leishmania using numerical zymotaxonomy Am J Trop Med Hyg. 1994;50(3): 296–311. 8147488 10.4269/ajtmh.1994.50.296

[51] Minodier P, Piarroux R, Gambarelli F, Joblet C, Dumon H. Rapid identification of causative species in patients with Old World leishmaniasis. J Clin Microbiol. 1997;35(10): 2551–2555. 9316906 10.1128/jcm.35.10.2551-2555.1997 PMC230009

[52] El Tai NO, Osman OF, el Fari M, Presber W, Schönian G. Genetic heterogeneity of ribosomal internal transcribed spacer in clinical samples of Leishmania donovani spotted on filter paper as revealed by single-strand conformation polymorphisms and sequencing. Trans R Soc Trop Med Hyg. 2000;94(5): 575–579. 11132393 10.1016/s0035-9203(00)90093-2

[53] Frank C, Hadziandoniou M, Pratlong F, Garifallou A, Rioux JA. *Leishmania tropica* and *Leishmania infantum* responsible for cutaneous leishmaniasis in Greece: sixteen autochthonous cases. Trans R Soc Trop Med Hyg. 1993;87: 184–185. 8337723 10.1016/0035-9203(93)90482-6

[54] Beltrame A, Arzese A, Camporese A, Rorato G, Crapis M, Tarabini-Castellani G et al Acute renal failure due to visceral leishmaniasis by Leishmania infantum successfully treated with a single high dose of liposomal amphotericin B. J Travel Med. 2008;15(5): 358–60. 10.1111/j.1708-8305.2008.00220.x 19006511

[55] Myrseli T, Velo E, Bino S, Alla L, Çomo N, Mersini K et al. Visceral and cutaneous leishmaniasis in Albania. Proc. of the 7th International Symposium on Phlebotomine Sand flies(ISOPS7), 25–30 Apr 2011, Kuşadasi, Turkey, P-024, p143.

[56] Portus M, Rioux J A, Gallego J, Lanotte G, Pratlong F, et al Les leishmanioses in Catalogne (Espagne). A propos de l’identification enzymatique de neuf souches d’origine humaine et canine In: Rioux JA editor. Leishmania. Taxonomie et Phylogenèse. Applications Eco-épideémiologiques. Montpellier: Institut Méditerranéen d’Etudes Epidemiologiques et Ecologiques 1986 pp. 433–438.

[57] Pratlong F, Rispail P, Moreno G, Le Fichoux Y, Tommasi C, Périères J et al Leishmania infantum MON-24 cutaneous leishmaniasis observed in Grassa (Alpes-Maritimes) in a Tunisian child. Ann Parasitol Hum Comp. 1989; 64(6): 506–509. 2624379 10.1051/parasite/1989646506

[58] Pratlong F, Rioux JA, Marty P, Faraut-Gambarelli F, Dereure J, Lanotte G et al Isoenzymatic Analysis of 712 Strains of *Leishmania infantum* in the South of France and Relationship of Enzymatic Polymorphism to Clinical and Epidemiological Features. J Clin Microb. 2004;42(9): 4077–4082. 10.1128/JCM.42.9.4077-4082.2004 PMC516332 15364993

[59] Portus M, Gallego J, Rioux JA, Pratlong F, Moreno G, Fisa R et al Enzymatic heterogeneity among strains of *Leishmania infantum* from human visceral and cutaneous leishmaniosis in Catalonia (Spain). Revista Iberica de Parasitologia.1989;49: 287–289.

[60] Pratlong F, Dedet JP, Marty P, Portús M, Deniau M, Dereure J et al Leishmania-Human Immunodeficiency Virus Coinfection in the Mediterranean Basin: Isoenzymatic Characterization of 100 Isolates of the *Leishmania infantum* Complex. J Infect Dis. 1995;172(1): 323–326. 7797943 10.1093/infdis/172.1.323

[61] Nimri L, Soubani R, Gramiccia M. Leishmania species and zymodemes isolated from endemic areas of cutaneous leishmaniasis in Jordan. Kinetoplastid Biol Dis. 2002;1(1): 7 12473179 10.1186/1475-9292-1-7 PMC149425

[62] Azmi K, Schnur L, Schonian G, Nasereddin A, Pratlong F, El Baidouri F et al Genetic, serological and biochemical characterization of *Leishmania tropica* from foci in northern Palestine and discovery of zymodeme MON-307. Parasit Vectors. 2012;5: 121 10.1186/1756-3305-5-121 22709680 PMC3432594

[63] Saliba E, Saleh N, Bisahrat Z, Oumeish O, Khoury S, Gramiccia M et al Cutaneous leishmaniasis due to *Leishmania tropica* in Jordan. Trans R Soc Trop Med Hyg. 1993;87: 633 8296360 10.1016/0035-9203(93)90267-t

[64] Gramiccia M, Scalone A, Di Muccio T, Orsini S, Fiorentino E, Gradoni L. The burden of visceral leishmaniasis in Italy from 1982 to 2012: a retrospective analysis of the multi-annual epidemic that occurred from 1989 to 2009. Euro Surveill. 2013; 18(29): 20535 23929120

[65] Gkolfinopoulou K, Bitsolas N, Patrinos S, Veneti L, Marka A, et al Epidemiology of human leishmaniasis in Greece, 1981–2011. Euro Surveill. 2013; 18(29): 20532 23929118

[66] Zeegelaar JE, Steketee WH, van Thiel PP, Wetsteyn JC, Kager PA, Faber WR. Changing pattern of imported cutaneous leishmaniasis in the Netherlands. Clin Exp Dermatol. 2005;30: 1–5. 15663490 10.1111/j.1365-2230.2004.01677.x

[67] Freedman DO, Weld LH, Kozarsky PE, Fisk T, Robins R, von Sonnenburg F et al Spectrum of disease and relation to place of exposure among ill returned travelers. N Engl J Med. 2006;354: 119–30. 16407507 10.1056/NEJMoa051331

[68] Lachaud L, Dedet J P, Marty P, Faraut F, Buffet P, Gangneux JP et al Surveillance of leishmaniases in France, 1999 to 2012. Euro Surveill. 2013;18(29): 38–44. 23929121

[69] Volf P, Benkova I, Myskova J, Sadlova J, Campino L, Ravel C. Increased transmission potential of *Leishmania major/Leishmania infantum* hybrids. Int J Parasitol. 2007;37: 589–593. 17376453 10.1016/j.ijpara.2007.02.002 PMC2839924

