# Correction: Epidemiology of Imported Leishmaniasis in Italy: Implications for a European Endemic Country

**DOI:** 10.1371/journal.pone.0134885

**Published:** 2015-07-31

**Authors:** Trentina Di Muccio, Aldo Scalone, Antonella Bruno, Massimo Marangi, Romualdo Grande, Orlando Armignacco, Luigi Gradoni, Marina Gramiccia

In the Funding section, the catalogue number from the EDENext Steering Committee is listed incorrectly. The correct number is: EDENext320.
